# Lessons for Understanding Central Nervous System HIV Reservoirs from the Last Gift Program

**DOI:** 10.1007/s11904-022-00628-8

**Published:** 2022-10-19

**Authors:** Patricia K. Riggs, Antoine Chaillon, Guochun Jiang, Scott L. Letendre, Yuyang Tang, Jeff Taylor, Andrew Kaytes, Davey M. Smith, Karine Dubé, Sara Gianella

**Affiliations:** 1 Department of Medicine, UCSD, San Diego, CA USA; 2 Department of Biochemistry and Biophysics, Institute of Global Health and Infectious Diseases, UNC HIV Cure Center, Chapel Hill, NC USA; 3 AntiViral Research Center (AVRC) Community Advisory Board, University of California San Diego, San Diego, CA USA; 4 HIV + Aging Research Project – Palm Springs (HARP-PS), Palm Springs, CA USA

**Keywords:** HIV reservoirs, Central nervous system (CNS), Cure, Eradication, Brain, Spinal cord

## Abstract

**Purpose of Review:**

Deep tissue HIV reservoirs, especially within the central nervous system (CNS), are understudied due to the challenges of sampling brain, spinal cord, and other tissues. Understanding the cellular characteristics and viral dynamics in CNS reservoirs is critical so that HIV cure trials can address them and monitor the direct and indirect effects of interventions. The Last Gift program was developed to address these needs by enrolling altruistic people with HIV (PWH) at the end of life who agree to rapid research autopsy.

**Recent Findings:**

Recent findings from the Last Gift emphasize significant heterogeneity across CNS reservoirs, CNS compartmentalization including differential sensitivity to broadly neutralizing antibodies, and bidirectional migration of HIV across the blood–brain barrier. Our findings add support for the potential of CNS reservoirs to be a source of rebounding viruses and reseeding of systemic sites if they are not targeted by cure strategies.

**Summary:**

This review highlights important scientific, practical, and ethical lessons learned from the Last Gift program in the context of recent advances in understanding the CNS reservoirs and key knowledge gaps in current research.

## Introduction

Within days of infection, HIV disseminates into the central nervous system (CNS) and throughout the body, establishing reservoirs of proviral DNA in infected cells [1, 2]. These HIV reservoirs persist indefinitely despite antiretroviral therapy (ART) and are the main obstacle to cure [1, 3•, 4–7]. Cure efforts have largely focused on CD4^+^ T cells in terms of characterizing the cellular reservoirs, therapeutic targets, and monitoring during analytical treatment interruption (ATI) [7, 8•]. However, the frequency of infiltration of T cells into the brain and their role in neuropathology is not fully understood. CD4+ and CD8+ T cells have been shown in brain tissue and CSF from people with and without HIV but further characterization is needed [cite 92, PMC6751344, PMC6214977]. In  addition, accumulating evidence has made it clear myeloid predominant and deep tissue reservoirs also need to be addressed as part of HIV cure strategies [8•, 9•, 10–13, 14••, 15]. These reservoirs continue to produce viral transcripts and proteins, which have been tied with local inflammation and pathology despite undetectable HIV RNA in blood [16–19]. Controversy exists about whether brain myeloid cells (BMCs) in protected reservoirs produce replication-competent HIV but current evidence supports that they do [7, 8•, 9•, 15, 16, 20–24]. This is important because, compared to lymphoid cells, BMCs are relatively resistant to the cytolytic effects of HIV as well as killing by cytotoxic T-lymphocytes (CTLs), which are a key component of many proposed cure strategies [14••, 25, 26]. These and other observations have increased interest in the mechanisms of persistence, viral dynamics, and strategies for targeting infected BMCs in deep-tissue reservoirs. In particular, the HIV reservoirs within the CNS are challenging to study due to limited access to brain and spinal tissues in living individuals. Some of key knowledge gaps in the current understanding of CNS reservoirs are summarized in Table 1.

**Table 1:** Key knowledge gaps for understanding CNS reservoirs guiding current and future investigations

What are the cellular characteristics of the CNS reservoirs and how do they differ within the CNS (especially CSF vs brain tissue)?
What are the molecular mechanisms contributing to persistence of HIV reservoirs and resistance to intrinsic and extrinsic cytolytic stimuli? How do these mechanisms differ between cell types (e.g., microglia vs T-cells) and immunologic microenvironments?
Do CNS reservoirs in humans have replication-competent provirus capable of rebound and systemic spread (proven in non-human primates but not yet in humans)?
How can cure therapies target CNS reservoirs? Should the intervention target only replication-competent provirus or all HIV transcription and translation in order to protect the CNS from neurotoxic viral proteins?
What are the direct and indirect effects of cure interventions on CNS reservoirs, CNS inflammation, microglial activation, inflammasome upregulation, neuronal injury, neurocognitive function, and mental health?
What is the effect of long-term suppressive ART on all of the above?

Effective ART has greatly altered the landscape of neurological complications due to HIV [27–29]. Two of the most severe complications, HIV-associated dementia (HAD) and HIV encephalitis, are now rare. However, the prevalence of mild-to-moderate HIV associated neurocognitive disorders (HAND) and mental health disorders has increased in people with HIV (PWH) [27–34]. Evidence that this may be driven by HIV reservoirs in the CNS and resulting unresolved neuroinflammation in the setting of suppressive ART includes associations between worse neurocognitive performance and higher HIV DNA levels in cerebrospinal fluid (CSF), CSF viral escape (detectable HIV RNA in CSF but not in blood), detection of Tat protein in CSF, and greater microglial activation based on Translocator Protein (TSPO) positron emission tomography (PET) imaging [19, 25, 35–38, 39••, 40, 41]. Notably, these do not require replication-competent provirus, underscoring the importance of even defective proviral genomes in injuring the brain, similarly to resting CD4^+^ T cells where immune activation could be triggered by defective HIV [42•, 43].

Most of the foundational data on neuro-HIV has been from the perspective of neuroinflammation and neuropathology, which relied on observational neuropsychological assessments, neurological examinations, neuroimaging, and blood and CSF biomarkers [16, 28, 34, 44, 35]. Organoid, animal, and especially non-human primate models have provided key insights into HIV reservoirs, viral dynamics, the immune response, and neurocognitive impairment [10, 14••, 44, 45–48]. Ongoing human research projects have collected autopsy tissues and created biobanks for more than two decades. These resources provide crucial biospecimens and linked data to determine the neuropathological correlates of disease [30, 49–54]. However, these existing projects were not specifically designed for HIV cure research, aiming for example to assess participants within months of death and perform autopsy within 24 h. Assessing participants’ viral loads and medications more frequently as death nears will allow improved antemortem phenotyping. Collecting tissues within 6 h of death better preserves cell viability and nucleic acid integrity, which will optimize use of newer technologies [55, 56]. The goal of the Last Gift cohort at the University of California San Diego (UCSD) is to accomplish this to better support HIV cure research, particularly focused on deep-tissue reservoirs [57–64].

## What Is the Last Gift Cohort?

The Last Gift is a unique cohort that enrolls altruistic PWH with a life-shortening illness who wish to participate in HIV cure research at the end-of-life (EOL), including tissue donation for a rapid research autopsy (completed within 6 h of death [65–67]), which greatly increases viable cell count and nucleic acid integrity [64, 68]).

The Last Gift cohort builds on the well-established institutional infrastructure and expertise of the California NeuroAIDS Tissue Network (CNTN) [69, 70]. Specifically, all participants in the Last Gift cohorts are co-enrolled in CNTN, which performs detailed neuropsychological testing, neurological examination, medical history (including ART history), and collection of blood and CSF at enrollment and then every 6 months. The Last Gift cohort expands the scope of CNTN in a subset of participants with close follow-up and sequential blood collection in the immediate antemortem period followed by a rapid research autopsy. These unique features make the Last Gift cohort a resource for ongoing and future HIV cure-related investigations using cutting-edge approaches that require viable cells or excellent RNA integrity, such as high dimensional multi-omics and single cell technologies, quantitative viral outgrowth assays (QVOA), next-generation sequencing (NGS), and cell sorting technologies.

The Last Gift rapid autopsy protocol also expands on standard CNTN protocols and includes collection of tissue samples from at least 40 different anatomic sites. The 16 CNS specific tissue samples collected are: basal ganglia, cerebellum, deep white matter, dura mater, frontal motor cortex, frontal premotor cortex, hippocampus, medulla, occipital cortex, pons, parietal cortex, prefrontal medial cortex, temporal cortex, and cervical, thoracic, and lumbar spinal cord. CSF is also collected from the right lateral ventricle. All brain tissue is collected from the right hemisphere so that the left hemisphere is preserved and stored per the CNTN protocol. To support a wide range of research methodologies, tissues are processed in several ways at time of rapid research autopsy. This includes specimens for Formalin-fixed paraffin embedded tissue for histological analysis, snap-frozen tissue collected in liquid nitrogen, preserved in RNALaterTM, as well as viable cell suspensions from each tissue. Several publications provide additional details on the methods [58, 59, 64, 65, 71, 72].

## Preliminary Data Generated from the Last Gift Study

Since June 2017, 31 participants have enrolled in the Last Gift cohort with 19 research autopsies completed, nearly all within 6 h of death. Nine participants remain in active follow-up, one participant withdrew, and two passed away without autopsy. Table 2 summarizes the demographic and disease characteristics for the 19 participants who completed the Last Gift protocol.

**Table 2:** Demographic and disease characteristics of the 19 participants who completed the Last Gift study with rapid research autopsy

Deceased with autopsy (*N* = 19)	*N* (%) or median [IQR]
Age: median [IQR]	63 [52–72]
Sex assigned at birth	
Male	17 (89.5%)
Female	2 (10.5%)
Gender	
Men	16 (84.2%)
Women	2 (10.5%)
Non-binary	1 (5.3%)
Race/ethnicity	
White/Non-Hispanic	15 (78.9%)
White/Hispanic	2 (10.5%)
Black/non-Hispanic	1 (5.3%)
Asian/Pacific Islander/non-Hispanic	1 (5.3%)
Continued ART until death	14 (73.7%)
Plasma HIV RNA < 50 copies/mL	15 (78.9%)
Last CD4 + T-cell count (cell/µL)	237 [138–464]
CD4 + T-cell Nadir (cell/µL)	63 [20–186]
Estimated duration of infection (years)	23 [15–31]
Terminal illness	
ALS	3 (15.8%)
Solid tumor malignancy	10 (52.6%)
Hematologic malignancy	2 (10.5%)
Other	4 (21%)

The initial analysis of the HIV reservoir characteristics and dynamics from the first six Last Gift participants were published just before the COVID-19 pandemic [57]. Throughout the pandemic, enrollment continued, and study assessments and rapid autopsies were performed with adjustments due to limited access to care facilities. In contrast, the virology and immunology labs experienced more disruption due to temporary closures and repurposing of resources and staff. As a result, most of the data summarized here will be from the initial six autopsies with an emphasis on CNS findings.

## CNS Reservoirs in the Last Gift

In the first six Last Gift rapid autopsies, HIV DNA levels were measured by digital droplet PCR (ddPCR) and full-length envelope single genome sequencing was performed on tissue and blood samples. Sequences were analyzed to determine phylogenetic relationships and bioinformatic approaches were used to track the dispersal of HIV DNA throughout the body. Two of these six participants discontinued ART prior to death and experienced antemortem viral rebound. The other four individuals remained virally suppressed on ART. The following sections organize findings based on the “Lessons Learned” from the investigations and summarized in Table 3.

**Table 3:** Summary of preliminary lessons and corresponding next steps for Last Gift and collaborators

Last Gift lessons about CNS reservoirs to date	Next steps
Heterogeneous distribution of HIV proviral DNA even within different cortical regions	1) Cell sorting, single-cell, and spatial omics analyses to determine cellular characteristics of the different reservoirs and immunologic microenvironments (microglial macrophage phenotypes, CD4 + subtypes) 2) Compare more CNS sites (multiple cortical areas, brain stem, spinal cord, and CSF)
CNS compartmentalization and differential bNAb susceptibility in brain tissue	1) Expand analysis with larger sample size and more CNS sites 2) Measure ART concentrations, resistance mutations 3) Tropism assays
Migration within brain and bidirectionally across BBB by HIV DNA sequence analysis and statistical modeling	1) Using single-cell techniques and spatial omics analyses to identify which cell types are migrating 2) Tropism
CNS has provirus with intact full length envelope gene, supportive of potential for viral rebound from CNS reservoirs	1) Adapted quantitative viral outgrowth assays, full-length HIV genome sequencing, and insertion site analysis to better characterize replication competence and clonality 2) Tropism of replication-competent virus

### Lesson Learned: the CNS Reservoirs Are Heterogeneous

**Fig. 1:**
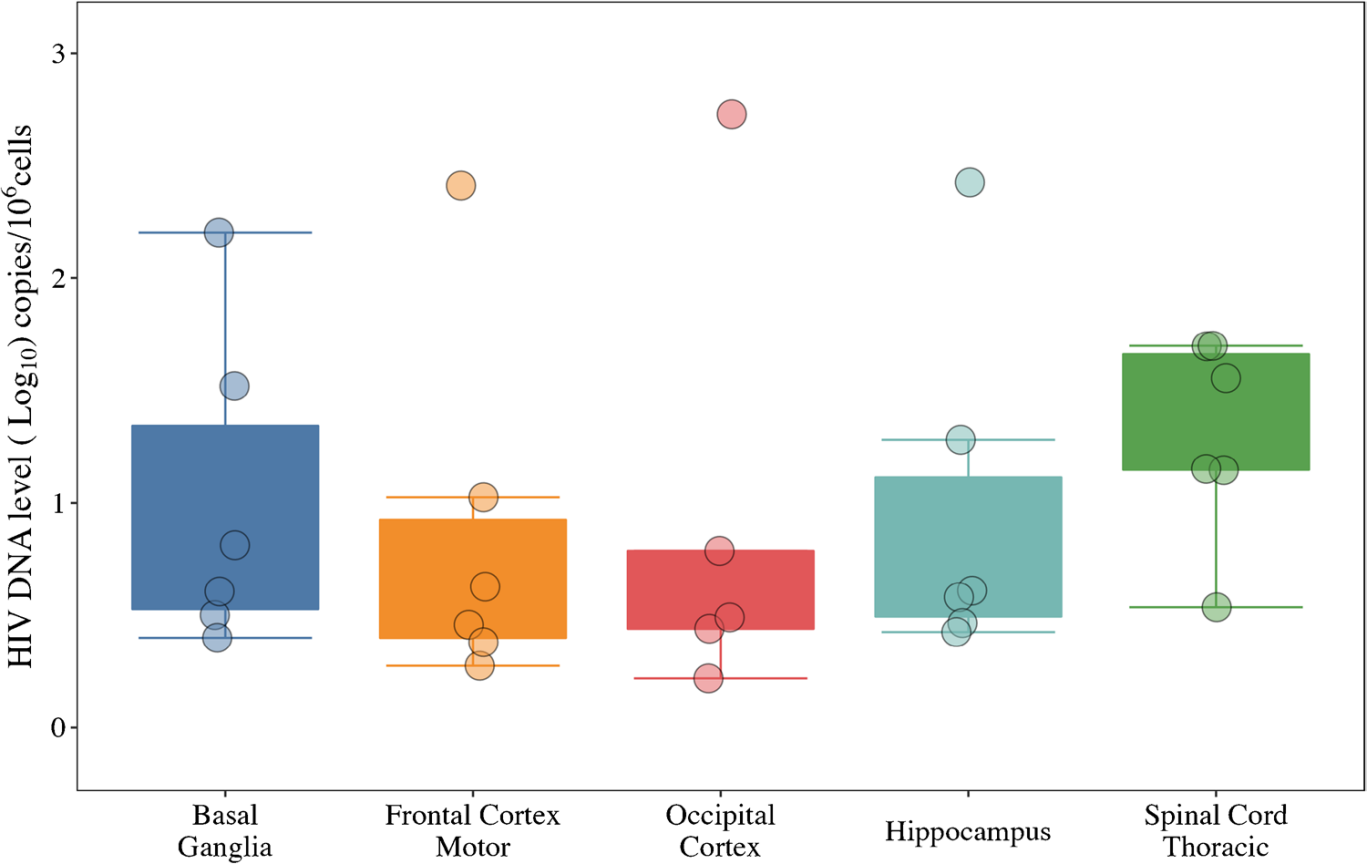
HIV DNA copies per 10^6^ cells by digital droplet PCR (ddPCR) in five CNS locations from the first six Last Gift participants

While the CNS reservoirs are often considered a single entity, this does not reflect the heterogeneity within the CNS. Overall, total HIV DNA levels in the CNS were lower than other anatomic sites, likely because the density of target cells is lower in the CNS than in lymphoid tissues. We detected HIV DNA within the CNS in all but one participant. Proviral DNA levels varied between CNS regions, with HIV DNA levels up to 4.6 copies per 10^6^ cells in the occipital lobe and up to 33.6 copies per 10^6^ cells in the frontal lobe. This is consistent with prior studies showing significant heterogeneity in the distribution of microglia ranging from 0.5–16.6% of all cells in the brain [48, 73, 74]. Due to the small sample size, initial analyses were not powered to determine a significant difference in distribution of HIV DNA within the CNS across individuals [6, 23, 57, 75, 76••], but HIV DNA levels were unexpectedly high in the spinal cord (see Fig. 1). Prior data on HIV DNA in spinal tissues is sparse [73, 77], but the findings strongly underscore the importance of sampling multiple sites simultaneously and the need for mapping the HIV reservoir across the CNS in relation to CD4+ T cells, microglia, astrocyte, and macrophage distribution [77]. It is interesting that two participants had amyotrophic lateral sclerosis, which has significant inflammation in the spinal cord, but these two samples were not outliers [78]. Initial analyses to estimate reservoir size focused on total HIV DNA (which includes both intact and non-intact provirus) and were performed on bulk brain tissue. Future studies will focus on the intact and replication-competent reservoir within cellular subsets.

Comparing the size and cellular characteristics of the HIV reservoir in the brain with CSF is also important because CSF is accessible and inexpensive to measure in living PWH and may serve as a window into deeper reservoir dynamics. The extent to which the CSF cellular reservoir reflects those in CNS tissues is largely unknown [25, 38, 39••, 40, 79, 80]. Prior studies have found that CSF contains HIV populations from both brain and systemic sources but most CSF cell-associated HIV DNA resides in T cells in contrast to the BMCs of deep brain tissues [39••, 81, 82]. Thus, analysis of paired specimens is underway and will advance understanding of the differences between CNS reservoirs, which will be important for HIV cure clinical trials and monitoring during analytical treatment interruptions.

### Lesson Learned: Some PWH Have CNS Compartmentalization and Distinct Susceptibility Patterns to Broadly Neutralizing Antibodies

**Fig. 2:**
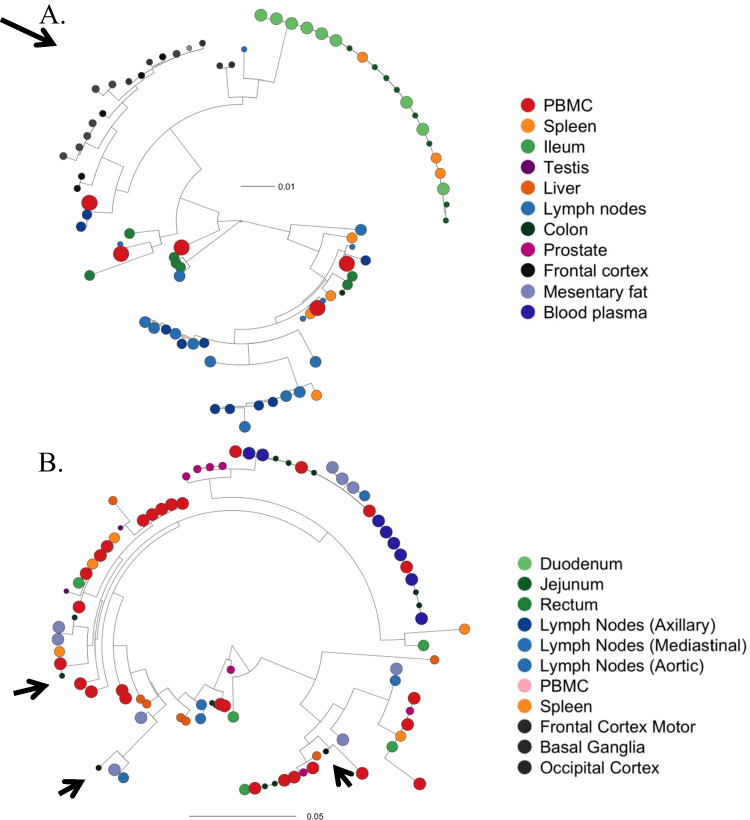
Maximum Likelihood (ML) Phylogenies and Clonal Populations (Full Length Envelope) for two participants who both remained virally suppressed at time of death. Phylogenies were estimated using IQtree [87] from the full length (FL) HIV env sequences obtained from ante-mortem blood plasma and from tissues and PBMC collected during rapid autopsy. Circle sizes reflect level of HIV DNA. **A** LG05 with pronounced viral compartmentalization within brain tissues. **B** LG08 without compartmentalization within CNS (arrows show frontal cortex). Both participants were heavily treatment-experienced with at least 8 years of suppressive ART. Figure adapted with permission from “HIV persists throughout deep tissues with repopulation from multiple anatomical sources.” By A. Chaillon, 2020, J Clin Invest, 130(4):1699–712

Statistical analysis confirmed evidence of compartmentalization of the HIV reservoirs populations in at least one anatomical site in all participants, including presence of CNS specific clades in some PWH, consistent with prior findings from CSF studies [25, 79, 80, 83–86]. Figure 2 shows viral phylogenies for two representative participants who were virally suppressed at time of death, one with CNS compartmentalization and one without. Autopsy-based analyses are unable to determine when compartmentalization occurred or confirm ongoing replication within the CNS prior to death while taking suppressive ART. However, it does confirm persistence of the distinct CNS reservoirs.

Prior studies have found HIV DNA has more antiretroviral resistance genes in the setting of CNS compartmentalization [88], possibly since CNS penetration of therapeutics is limited by the blood brain barrier to varying degrees. Levels also vary significantly between CNS sites particularly between CSF and brain tissue [89, 90]. Others have shown transition to more X4-tropic virus in the CNS with compartmentalization and later in disease course [91, 92]. Therefore, CNS compartmentalization likely reflects differential selective pressure and adaptation to BMCs [80, 91]. Our initial sample size was too small to replicate these findings, but we will further investigate tropism and CNS ART levels.

It is not yet clear whether compartmentalization will be an important distinction beyond the presence of a persistent CNS reservoir for cure strategies and is likely to depend on the type of intervention. Recently reported data started to address this issue focusing on tissue-specific HIV neutralization by broadly neutralizing antibodies. Using mathematical modeling, our group found that of all tissues analyzed, brain tissue was the only tissue with significantly different predicted susceptibility patterns to 9 bNAbs currently in cure trials or under development. Brain tissue bNAb predicted susceptibilities differed significantly from blood/PBMCs, lymphatic and gastrointestinal tissues (*p*-values: 0.002, 0.0008, and 0.03, respectively) [93]. Together, our data provide further evidence that infected cells can survive within the brain for extended periods of time and that they might have unique characteristics that should be considered when designing HIV cure interventions.

### Lesson Learned: CNS Reservoirs are Dynamic with Migration Within the CNS and Bidirectionally Across the Blood Brain Barrier

**Fig. 3:**
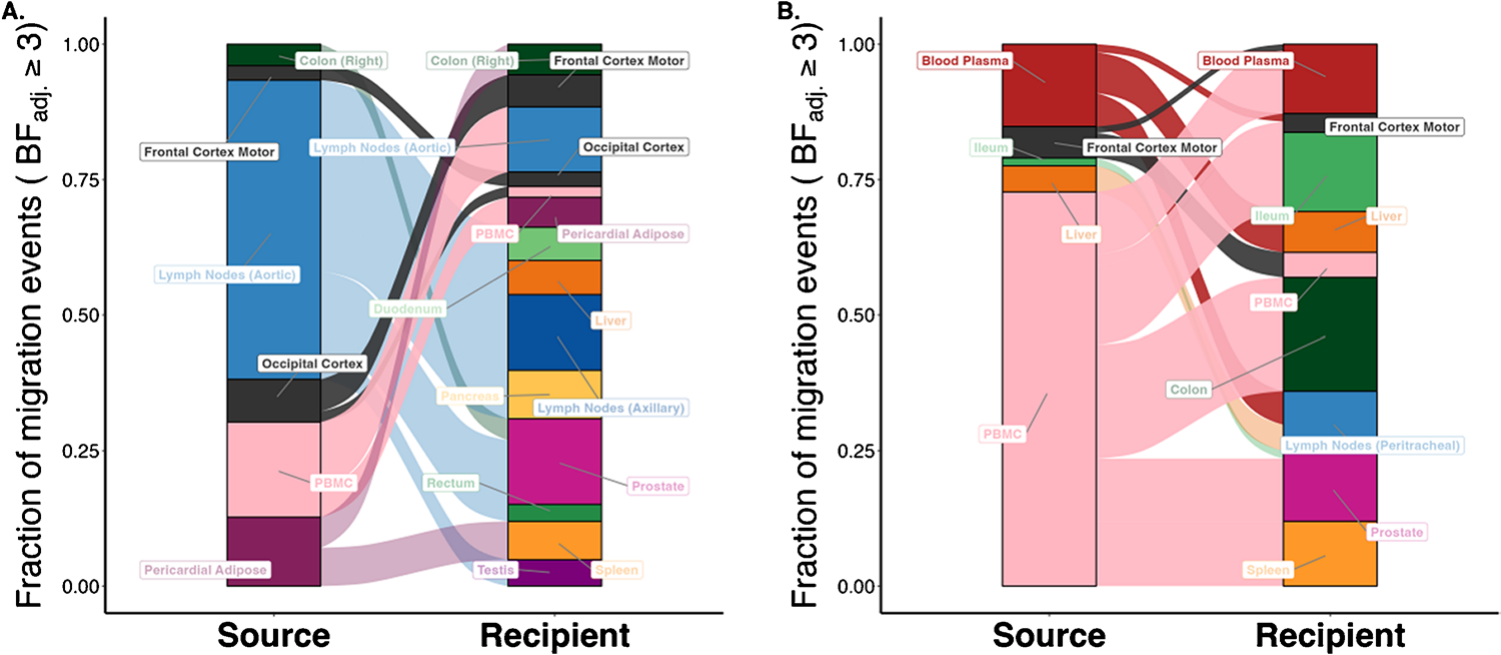
Sankey plots showing the proportion of migration events between anatomic locations for which the adjusted Bayes factor (BF) was 3 or higher for two participants showing migration within brain sites and bidirectionally across the blood brain barrier. The source locations are depicted on the left side of the plots and the destination locations on the right side. **A** Participant LG03 who remained virally suppressed on ART. Plots show evidence of bidirectional migration events of HIV DNA between the frontal motor and occipital cortex and occipital cortex to peripheral blood mononuclear cells [PBMCs]. **B** Data for a participant LG01 who stopped ART 53 days prior to death with viral rebound (antemortem plasma HIV RNA of 280 copies/mL). Note transitions of HIV DNA from frontal motor cortex to PBMC and plasma (and vice versa). Figure adapted with permission from “HIV persists throughout deep tissues with repopulation from multiple anatomical sources.” By A. Chaillon, 2020, J Clin Invest, 130(4):1699–712

The Last Gift project used novel statistical approaches to track dispersal patterns of HIV DNA across tissues [57, 72]. Overall, migration events were more common between tissues within the same biological system (e.g., CNS, gastrointestinal systems) but were not limited by anatomic location or organ system. Figure 3 illustrates migration patterns Inferred from Bayesian discrete trait analyses for two representative participants demonstrating dynamic transitions within the cortex and bidirectionally between the cortical sites and systemic blood and PBMCs. This demonstrates the ability of HIV DNA, and most likely infected cells, to migrate across the brain and from the brain to systemic circulation supporting the possibility of viral rebound in the CNS leading to reseeding of systemic reservoirs [57, 80, 85].

Interestingly, there was no correlation between the HIV DNA levels in a tissue and the number of migration events. This is further evidence that the relatively low levels of proviral DNA in the CNS tissues does not necessarily correspond to the level of risk of migration from the CNS. Additional work is ongoing to determine the cellular subtypes that traffic to other sites and to evaluate replication competence.

### Lesson Learned: Challenges Remain in Definitive Evaluation of the Potential for Viral Rebound and Dissemination from CNS Reservoirs

Production of replication-competent HIV solely from CNS-resident cells has not yet been definitively proven in humans largely due to methodological limitations. The strongest evidence for replication competent provirus in microglia comes from non-human primate (NHP) using macrophage QVOA studies showing replication-competent HIV provirus in microglia despite ART [4, 13, 14••]. NHP models are highly valuable but findings from NHP studies are not always confirmed in human studies [94–99]. A key limitation in NHP models is the relatively short duration of SIV infection and ART that is feasible in NHPs compared to human. This is important for evaluating the reservoir sizes and percentage of intact or replication-competent proviral DNA considering prior evidence that viral decay is most affected by ongoing replication, not the initial viral set point [5, 100, 101].

Another key difficulty in directly proving potential for viral rebound and systemic spread from CNS reservoirs is that detection of HIV DNA or RNA is not a surrogate for replication competence (or intact proviral genome). The majority of HIV DNA is either inert or produces viral transcripts and proteins but not replication-competent virus. The gold standard to determine replication competence has been QVOA but it requires many viable cells and significant time and expense even though it underestimates the size of the replication-competent reservoir [3•]. Additionally, QVOA was developed for ex vivo stimulation of viral replication in T cells and was only recently adapted for macrophages [8•, 13, 14••]. NGS technology can overcome many of the described limitations of QVOA for tissue specimens [3•], allowing assessment of intact proviral genomes and evaluation of the relative viral diversity in blood and in tissues but is currently cost and resource prohibitive for large scale use. The commercially available intact proviral DNA assay (IPDA) has shown promise in measuring intact provirus but also needs significant amount of cells, is clade-dependent and does not provide sequence data [102].

For our initial analysis of the first six participants, up to 90% (range 43–90%) of HIV proviruses recovered from CNS tissues were intact based on full-length envelope sequencing [57], which is higher than prior animal and autopsy studies [103–105]. This is likely an overestimate since we only focused on one HIV gene (*env*). Since genomic DNA is more stable postmortem than RNA, it is unlikely that this simply reflects the better yield due to rapid autopsy. The next step for the Last Gift project is to perform full-length genome sequencing and integration site analysis together with other methods of quantifying intact provirus and replication-competence [3•, 106•].

### Lesson Learned: Cohort Heterogeneity Can Be a Gift and a Challenge

Last Gift participants would generally be excluded from research due to the confounding inherent in their non-AIDS defining terminal illnesses. However, it is fitting for this era of personalized medicine and focus on what the individual or outliers within study samples can teach us [107–111]. In fact, looking at the HIV cure research field, the major milestones have been successful cure or HIV ART free remission in one person at a time, starting with Timothy Ray Brown (the “Berlin Patient”) in 2008 [21, 112, 113]. The range of pathologies in Last Gift participants likely influences the HIV reservoir, particularly in diseased tissues. Pathologies and treatments for these diseases (e.g., cancer and chemotherapy) are accounted for to the degree possible. For example, we collected and stored the brain of a person with multiple brain lesions but excluded it from virological analyses of the CNS reservoir.

We are currently generating longitudinal biomarkers to evaluate organ dysfunction, inflammation, gut dysbiosis during the peri-mortem periods, and we will evaluate how these conditions might affect the HIV reservoirs at the time of death. Despite these limitations, Last Gift participants are uniquely valuable for assessment of patterns across participants, such as tissue-specific viral phylogenies. Unexpected insights can also result from the research. For example, some of the data on T cell clonal expansion and maintenance of reservoirs were initially generated from samples taken to evaluate tumor microenvironments and found enrichment of HIV-infected T cells surrounding the tumor [106•, 114]. The Last Gift may similarly provide insights into characteristics of interactions between HIV and comorbid conditions, including those that affect the CNS. Even if no clear patterns of HIV persistence are identified, the Last Gift program will still represent a comprehensive and unprecedented characterization of blood and tissue HIV reservoirs, which will be a valuable resource for future investigations.

## Practical Lessons Learned from the Last Gift Study

The field of HIV cure research needs more studies similar to the Last Gift and we wish to encourage and facilitate this. Since the study brought many new challenges for us, we wish to share some general lessons learned for other teams interested in pursuing similar studies.

### Lesson Learned: EOL and Rapid Autopsy for HIV Research Is Feasible

#### EOL Research Is Feasible

As the first study with rapid autopsy in the field of HIV research, one of the most important lessons is that this can be done in a reliable and ethical manner. Rapid autopsy studies in other fields generally had more limited tissue collection (e.g., collection of tumor and regional lymph nodes) and the number of tissues collected and processed within 6 h for Last Gift participants is unprecedented to our knowledge. Overall, research autopsy time has shortened with experience in managing the legal, logistical, and procedural processes both before and immediately after death.

An additional concern was that rapid autopsy tissue from people who died of natural causes is not directly analogous to necropsy in animal research or tissue samples collected through biopsies from living individuals. For example, the physiologic process of gradual human death could alter microglial phenotypes, viral transcription, and viral dynamics in unique ways compared to animal euthanasia [42•, 115–117]. It is unknown whether CNS cells, particularly BMCs that are considered the major HIV reservoirs in the CNS, are viable 6 h post-mortem and whether the quality of those isolated BMCs are good enough to determine the replication competent HIV ex vivo. However, so far with our protocols for cell sorting and isolation of BMCs, including microglia and macrophages, up to 10^6^ viable BMCs/gram of fresh brain tissues have been reliably collected. These cells are IBA1 + , CD68 + , and TMEM119 + , which retain HIV DNA/RNA and produce outgrowth HIV after latency reversal (unpublished data). Studies are ongoing to further determine the replication-competent HIV and the underlying mechanisms of HIV latency in those BMCs ex vivo.

### Lesson Learned: EOL and Rapid Autopsy Studies Pose Unique Challenges

#### Study Flexibility

Basic components of study design are different in EOL and rapid autopsy research compared to traditional studies with predetermined visit schedules and duration of enrollment. For example, the visit schedule needs flexibility to account for participant’s clinical condition and is essentially trying to count backward from the estimated time of death which, of course, is difficult to predict due to prognostic uncertainty. This means that the first three study visits may occur over 9 months or within one week since the goal is to have frequent, brief visits with HIV RNA results as close to death as possible. This represents a paradigm shift to individualize visit schedules with implications from database set-up, informed consent, and study visits. Similarly, the unpredictability of antemortem events requires more frequent communication with participants, caregivers, and next-of-kin/loved ones and round-the-clock availability of study staff.

#### Study Visits at Home or Facility

The visits near the EOL are not conducted at the study site. Study staff travel to the participants’ home or healthcare facility. This is a necessity but does raise many legal and logistical hurdles to ensure legality of conducting research off-site, which need to be addressed with a memorandum of understanding and signed by each party involved. If a participant is in a facility, substantial documentation and reminders are needed about study participation and procedures at the time of death.

#### Phlebotomy at the End of Life

One major challenge is that blood collection frequently becomes more technically difficult closer to death due to edema and hypovolemia. Also, most participants are on hospice or comfort care near death and are unlikely to have central venous catheters or clinical reasons for phlebotomy. Employing experienced phlebotomists is critical.

#### Tissue Analysis Versus Fluid Samples

Analysis of tissue introduces additional difficulties compared with cells or extracellular fluid from body fluids, in part because of its three-dimensional architecture. Decades of clinical and research assays have been primarily based on body fluids and adapting tests to solid tissues is challenging. Tissues can be homogenized but can also be contaminated with blood, even small amounts of which may affect findings [61]. However, the impact is likely to be a small considering the small size of capillaries compared to overall tissue mass. Furthermore, tissue-resident lymphocytes have immune profiles distinct from circulating lymphocytes [118], which we will assess in our bulk expression analyses [119, 120] to exclude tissues heavily contaminated with blood. Variability in the number of immune cells in tissues could also contribute to variation in HIV levels. To normalize HIV DNA copy counts in tissues, we use published methods to quantify CD4^+^ T-cell numbers in tissue by ddPCR [121] although pre-sorting various population will provide the most information.

#### Shipping Specimens

Death does not always occur during business hours. While the study team is on call and prepared to work at any time, we still rely on logistical services. One challenge is shipping biospecimens to collaborators at any time, day, or night. This requires coordination with the receiving labs outside of their regular hours and use of alternative shipping companies, which can lead to higher prices.

### Lesson Learned: the Critical Importance of Multi-disciplinary Research Teams and Community Involvement

Developing the Last Gift cohort has underscored the critical importance of multi-disciplinary, translational research teams with meaningful and sustained community involvement and engagement. This goes beyond “bench to bedside” and includes bioethics, socio-behavioral sciences. Key lessons summarized in Table 4.

**Table 4:** Key practical and ethical lessons learned – end of life and rapid autopsy research

Research at EOL has provided our participants with autonomy and ability to leave a meaningful scientific legacy
Sustained community engagement and patient/participant-centered focus is particularly critical in EOL research
Multi-disciplinary teams are key and should include paid positions for socio-behavioral scientists and members of the HIV community
Decisions regarding goals-of-care, hospice, and medical aid in dying (where applicable) must be done completely outside the study

#### Bioethics

At inception, we sought bioethics expertise to establish detailed ethical considerations for HIV reservoir research at the EOL [122, 123]. We emphasized five ethical domains: (1) protection of autonomy through informed consent, (2) avoidance of exploitation and fostering of altruism, (3) maintenance of favorable benefits/risks, (4) safeguarding against vulnerability through patient/participant-centeredness, and (5) acceptance of research by next-of-kin/loved ones and community. As the Last Gift program unfolded, we identified, documented, resolved, and integrated ethical lessons learned. For example, due to the challenge of declining cognitive function at the EOL, our team has implemented process consent. We test cognition multiple times during the study to ensure participants continue to consent to research procedures (e.g., blood draws) and the tissue donation as they approach the EOL [122]. With rapid research autopsy focused on the brain region, we attempt to minimize the degree of invasiveness of the research and justify in terms of expected scientific benefits. In particular, we minimize the risk of unanticipated disfigurement and preserve dignity of person after their passing, consistent with ethics guidelines with the recently dead [124]. Furthermore, while Last Gift participants can elect to follow medical aid in dying (MaiD) now legal in California, this decision and process must be done completely outside the scope of the Last Gift [59, 122, 125].

The next research frontier will be evaluating the effects of interventions on the CNS and deep tissue compartments. Our team is proactively evaluating ethical considerations of interventional HIV cure-related research at the EOL [123]. As with all clinical research, we have an ethical obligation to limit risk of undue pain, suffering, and accelerated death at the end of life [123]. Only well-vetted interventions supported by robust pre-clinical data should be considered for testing at the EOL [123].

#### Socio-Behavioral Sciences

The Last Gift program has employed a 360-degree approach to socio-behavioral sciences to understand perspectives and experiences of participants [126], next-of-kin/loved ones [71, 127], clinical and rapid research autopsy staff [60], community members, and other stakeholders. This holistic approach helps ensure sensitive EOL research remains acceptable to various groups. The Last Gift team must continue to understand how PWH make decisions to participate in HIV reservoir research at the end of life. This is critical to fully informed research designs that preserve participants’ voices and lived experiences. More empirical research will be needed to understand the mental health, emotional, psychosocial, and social determinant aspects of EOL research participation.

#### Meaningful Community Involvement and Engagement

The HIV community has been crucial in designing and implementing the Last Gift study [128–130]. The Last Gift concept was initially controversial with some reviewers and the HIV research community, which raised concerns about “vulnerability.” However, PWH and community advocates fiercely defended the Last Gift idea [128] and advised basic and biomedical scientists from the beginning. This involvement was critical in ensuring mutual understanding and alignment of research goals and priorities. Due to age, co-morbidities, and longer duration of HIV infection prior to ART [131], HIV long-term survivors have been excluded from HIV cure research, often after being at the forefront of lifesaving HIV discoveries in the 1980s and 1990s. The Last Gift program gives them an unprecedented opportunity to contribute and leave a legacy (“last gift”) to the community. For example, one issue arose where an institutional review board viewed cremation as a study benefit; however, community advocates requested it be considered a necessity given the nature of research. Community members came up with the name ‘On Deck’ for potential participants who did not meet the 6-month prognosis inclusion criteria but wanted to be considered for the Last Gift in due course. The puzzle-piece logo for Last Gift was also designed by a community representative to symbolize how each study participant is a piece of a larger puzzle toward an HIV cure and ending the HIV epidemic. Community members have continued to advise on several aspects the Last Gift program.

Furthermore, the Last Gift adopts an inclusive approach with next-of-kin/loved ones, who play an integral role in EOL research [71, 127]. We learned that early involvement of and clear communication with next-of-kin/loved ones are necessary. More research will be needed to understand perspectives of less supportive next-of-kin/loved ones [127]. To preserve public acceptance and trustworthiness [132] of EOL research, sustained community engagement is essential. The Last Gift has a strong commitment to equity, diversity, and inclusion, and understanding how cultural and spiritual differences affect willingness to participate. We are also working to increase diversity in the cohort in order to study how sex, race, and ethnicity affect HIV reservoir and CNS dynamics as this continues to remain a key research gap [59, 63, 133, 134].

## Conclusions

The possibility of a cure for HIV holds the potential for individuals to avoid or reduce the effects of chronic HIV, ART toxicity, polypharmacy, treatment expense (when compared to the cost of ART over a lifetime), HIV transmission, and stigma, among many others. Many possibilities are under investigation that would present safer and more feasible options for the larger population of PWH, other than stem cell transplants for participants with concomitant hematologic malignancies. As the management of HIV continues to become increasingly well tolerated and effective, the level of acceptable risk of adverse effects for HIV cure trials decreases. Thus, cure scientists must make the best use of each clinical trial opportunity, which heightens the importance of fully characterizing the diverse HIV reservoirs in the brain, spinal cord, and other organs. The Last Gift, and similar studies, will be a critical piece of this larger puzzle and complement the other investigations.

So far, the Last Gift program has provided important insights into the CNS reservoirs and continues to do so in innovative ways. Our preliminary data showed migration of HIV DNA from brain tissues to systemic circulation and that migration events are not proportional to the size of the reservoir. Additional studies are underway but our findings add to the evidence of the risk of viral rebound from CNS reservoirs if cure strategies do not specifically target them. Also, the discovery that CNS reservoirs in brain may have different bNAb susceptibilities than blood and other deep reservoirs has direct implications for cure trial.

The past two decades have taught us that sustained viral suppression in blood and even CSF is not enough to avoid neurologic complications from HIV. Sustained viral suppression in blood and CSF is an essential, but not sufficient measure of successful ART-free remission. If HIV is eradicated or silenced within the blood and PBMCs, we need to ensure there is not ongoing viral transcription and associated inflammation in deeper, less accessible reservoirs. The clinical impact of such local inflammation is not known but is reasonable to anticipate it may be subtle and gradual. Therefore, monitoring for known adverse effects of the cure therapies and unstructured, subjective reporting of mood or neurocognitive changes is not sufficient. Biomarkers and neuroimaging may provide additional information but cannot replace neurocognitive testing and standard psychiatric screening questionnaires. We recognize that it will be difficult to find the critical balance between undue participant burden, study expense and appropriate monitoring but argue that the field cannot afford to miss potential adverse effects. This will continue to require the innovation, dedication, advocacy, and community partnerships that have defined the last four decades of HIV research.

Additionally, due to limitations in geographic reach of an individual study site, need for larger sample sizes with more diverse populations, the field will be best served by multiple collaborative sites doing similar studies. It is a large undertaking for the number of participants, however, the amount of data from each participant is extraordinary and adds social and scientific value. In addition, the inclusion of community members as part of the study team has reinvigorated the bond between people with HIV and researchers. Even more, the privilege of working with truly remarkable individuals at the end of their life is profoundly meaningful for the entire team.

## References

[1] Abrahams MR, Joseph SB, Garrett N, Tyers L, Moeser M, Archin N, et al. The replication-competent HIV-1 latent reservoir is primarily established near the time of therapy initiation. Sci Transl Med. 2019;11(513):eaaw5589. 10.1126/scitranslmed.aaw5589 PMC7233356 31597754

[2] Valcour V, Chalermchai T, Sailasuta N, Marovich M, Lerdlum S, Suttichom D (2012). Central nervous system viral invasion and inflammation during acute HIV infection. J Infect Dis.

[3.•] Abdel-Mohsen M, Richman D, Siliciano RF, Nussenzweig MC, Howell BJ, Martinez-Picado J (2020). Recommendations for measuring HIV reservoir size in cure-directed clinical trials. Nat Med.

[4] Avalos CR, Abreu CM, Queen SE, Li M, Price S, Shirk EN, et al. Brain macrophages in simian immunodeficiency virus-infected, antiretroviral-suppressed macaques: a functional latent reservoir. mBio. 2017;8(4):e01186-17. 10.1128/mBio.01186-17 PMC5559639 28811349

[5] Bachmann N, von Siebenthal C, Vongrad V, Turk T, Neumann K, Beerenwinkel N (2019). Determinants of HIV-1 reservoir size and long-term dynamics during suppressive ART. Nat Commun.

[6] Churchill MJ, Deeks SG, Margolis DM, Siliciano RF, Swanstrom R (2016). HIV reservoirs: what, where and how to target them. Nat Rev Microbiol.

[7] Henderson LJ, Reoma LB, Kovacs JA, Nath A. Advances toward curing HIV-1 infection in tissue reservoirs. J Virol. 2020;94(3):e00375-19. 10.1128/JVI.00375-19 PMC7000960 31694954

[8.•] Mitchell BI, Laws EI, Ndhlovu LC (2019). Impact of myeloid reservoirs in HIV cure trials. Curr HIV/AIDS Rep.

[9.•] Chan P, Ananworanich J (2019). Perspective on potential impact of HIV central nervous system latency on eradication. AIDS.

[10] Honeycutt JB, Wahl A, Baker C, Spagnuolo RA, Foster J, Zakharova O (2016). Macrophages sustain HIV replication in vivo independently of T cells. J Clin Invest.

[11] Honeycutt JB, Thayer WO, Baker CE, Ribeiro RM, Lada SM, Cao Y (2017). HIV persistence in tissue macrophages of humanized myeloid-only mice during antiretroviral therapy. Nat Med.

[12] Cenker JJ, Stultz RD, McDonald D (2017). Brain microglial cells are highly susceptible to HIV-1 infection and spread. AIDS Res Hum Retroviruses.

[13] Avalos CR, Price SL, Forsyth ER, Pin JN, Shirk EN, Bullock BT (2016). Quantitation of productively infected monocytes and macrophages of simian immunodeficiency virus-infected macaques. J Virol.

[14.••] Abreu C, Shirk EN, Queen SE, Beck SE, Mangus LM, Pate KAM (2019). Brain macrophages harbor latent, infectious simian immunodeficiency virus. AIDS.

[15] Winston A, Julie F, Fidler S (2017). HIV cure strategies: response to ignore the central nervous system at your patients’ peril. AIDS.

[16] Lv T, Cao W, Li T (2021). HIV-related immune activation and inflammation: current understanding and strategies. J Immunol Res.

[17] Montoya JL, Campbell LM, Paolillo EW, Ellis RJ, Letendre SL, Jeste DV (2019). Inflammation relates to poorer complex motor performance among adults living with HIV on suppressive antiretroviral therapy. J Acquir Immune Defic Syndr.

[18] Zicari S, Sessa L, Cotugno N, Ruggiero A, Morrocchi E, Concato C (2019). Immune activation, inflammation, and non-AIDS co-morbidities in HIV-infected patients under long-term ART. Viruses.

[19] van Zoest RA, Underwood J, De Francesco D, Sabin CA, Cole JH, Wit FW (2017). Structural brain abnormalities in successfully treated HIV infection: associations with disease and cerebrospinal Fluid Biomarkers. J Infect Dis.

[20] Li GH, Henderson L, Nath A (2016). Astrocytes as an HIV reservoir: mechanism of HIV infection. Curr HIV Res.

[21] Ding J, Liu Y, Lai Y (2021). Knowledge from London and Berlin: finding threads to a functional HIV cure. Front Immunol.

[22] Sengupta S, Siliciano RF (2018). Targeting the Latent Reservoir for HIV-1. Immunity.

[23] Dufour C, Gantner P, Fromentin R, Chomont N (2020). The multifaceted nature of HIV latency. J Clin Invest.

[24] Farber DL (2021). Tissues, not blood, are where immune cells function. Nature.

[25] Gisslen M, Keating SM, Spudich S, Arechiga V, Stephenson S, Zetterberg H (2021). Compartmentalization of cerebrospinal fluid inflammation across the spectrum of untreated HIV-1 infection, central nervous system injury and viral suppression. PLoS ONE.

[26] Kearney MF, Wiegand A, Shao W, Coffin JM, Mellors JW, Lederman M (2016). Origin of rebound plasma HIV includes cells with identical proviruses that are transcriptionally active before stopping of antiretroviral therapy. J Virol.

[27] Yuan NY, Kaul M (2021). Beneficial and adverse effects of cART affect neurocognitive function in HIV-1 infection: balancing viral suppression against neuronal stress and injury. J Neuroimmune Pharmacol.

[28] Letendre S (2011). Central nervous system complications in HIV disease: HIV-associated neurocognitive disorder. Top Antivir Med.

[29] Lin SP, Calcagno A, Letendre SL, Ma Q (2021). Clinical treatment options and randomized clinical trials for neurocognitive complications of HIV infection: combination antiretroviral therapy, central nervous system penetration effectiveness, and adjuvants. Curr Top Behav Neurosci.

[30] Gutierrez J, Byrd D, Yin MT, Morgello S (2019). Relationship between brain arterial pathology and neurocognitive performance among individuals with human immunodeficiency virus. Clin Infect Dis.

[31] Lanman T, Letendre S, Ma Q, Bang A, Ellis R (2021). CNS neurotoxicity of antiretrovirals. J Neuroimmune Pharmacol.

[32] Mackiewicz MM, Overk C, Achim CL, Masliah E (2019). Pathogenesis of age-related HIV neurodegeneration. J Neurovirol.

[33] Fernandes N, Pulliam L. Inflammatory mechanisms and cascades contributing to neurocognitive impairment in HIV/AIDS. Berlin: Springer Berlin Heidelberg. p. 1–27. 10.1007/7854_2019_100 31385260

[34] Heaton RK, Clifford DB, Franklin DR, Woods SP, Ake C, Vaida F (2010). HIV-associated neurocognitive disorders persist in the era of potent antiretroviral therapy: CHARTER Study. Neurology.

[35] Akiyama H, Jalloh S, Park S, Lei M, Mostoslavsky G, Gummuluru S. Expression of HIV-1 intron-containing RNA in microglia induces inflammatory responses. J Virol. 2020:e01386-20. 10.1128/JVI.01386-20 PMC8092841 33298546

[36] Chivero ET, Guo M-L, Periyasamy P, Liao K, Callen SE, Buch S (2017). HIV-1 Tat primes and activates microglial NLRP3 inflammasome-mediated neuroinflammation. J Neurosci.

[37] de Oliveira MF, Gianella S, Letendre S, Scheffler K, Kosakovsky Pond SL, Smith DM (2015). Comparative analysis of cell-associated HIV DNA levels in cerebrospinal fluid and peripheral blood by droplet digital PCR. PLoS ONE.

[38] Winston A, Antinori A, Cinque P, Fox HS, Gisslen M, Henrich TJ (2019). Defining cerebrospinal fluid HIV RNA escape: editorial review AIDS. AIDS.

[39.••] Joseph SB, Kincer LP, Bowman NM, Evans C, Vinikoor MJ, Lippincott CK (2019). Human immunodeficiency virus type 1 RNA detected in the central nervous system (CNS) after years of suppressive antiretroviral therapy can originate from a replicating cns reservoir or clonally expanded cells. Clin Infect Dis.

[40] Spudich S, Robertson KR, Bosch RJ, Gandhi RT, Cyktor JC, Mar H (2019). Persistent HIV-infected cells in cerebrospinal fluid are associated with poorer neurocognitive performance. J Clin Investig.

[41] Werry EL, Bright FM, Piguet O, Ittner LM, Halliday GM, Hodges JR, et al. Recent developments in TSPO PET imaging as a biomarker of neuroinflammation in neurodegenerative disorders. Int J Mol Sci. 2019;20(13):3161. 10.3390/ijms20133161 PMC6650818 31261683

[42.•] Musick A, Spindler J, Boritz E, Perez L, Crespo-Velez D, Patro SC (2019). HIV Infected T cells can proliferate in vivo without inducing expression of the integrated provirus. Front Microbiol..

[43] Pollack RA, Jones RB, Pertea M, Bruner KM, Martin AR, Thomas AS (2017). Defective HIV-1 proviruses are expressed and can be recognized by cytotoxic T lymphocytes, which shape the proviral landscape. Cell Host Microbe.

[44] Gumbs SBH, Kubler R, Gharu L, Schipper PJ, Borst AL, Snijders G, et al. Human microglial models to study HIV infection and neuropathogenesis: a literature overview and comparative analyses. J Neurovirol. 2022:64–91. 10.1007/s13365-021-01049-w PMC9076745 35138593

[45] Moretti S, Virtuoso S, Sernicola L, Farcomeni S, Maggiorella MT, Borsetti A. Advances in SIV/SHIV Non-Human Primate Models of NeuroAIDS. Pathogens. 2021;10(8):1018. 10.3390/pathogens10081018 PMC8398602 34451482

[46] Carryl H, Swang M, Lawrence J, Curtis K, Kamboj H, Van Rompay KK, et al. Of mice and monkeys: can animal models be utilized to study neurological consequences of pediatric HIV-1 infection? ACS Chem Neurosci. 2015;6(8):1276–89. 10.1021/acschemneuro.5b00044 PMC4545399 26034832

[47] North TW, Higgins J, Deere JD, Hayes TL, Villalobos A, Adamson L (2010). Viral sanctuaries during highly active antiretroviral therapy in a nonhuman primate model for AIDS. J Virol.

[48] Lawson L, Perry V, Gordon S (1992). Turnover of resident microglia in the normal adult mouse brain. Neuroscience.

[49] Soontornniyomkij V, Umlauf A, Soontornniyomkij B, Gouaux B, Ellis RJ, Levine AJ (2018). Association of antiretroviral therapy with brain aging changes among HIV-infected adults. AIDS.

[50] Soontornniyomkij V, Moore DJ, Gouaux B, Soontornniyomkij B, Sinsheimer JS, Levine AJ (2019). Associations of regional amyloid-beta plaque and phospho-tau pathology with biological factors and neuropsychological functioning among HIV-infected adults. J Neurovirol.

[51] Umlauf A, Soontornniyomkij B, Sundermann EE, Gouaux B, Ellis RJ, Levine AJ (2019). Risk of developing cerebral beta-amyloid plaques with posttranslational modification among HIV-infected adults. AIDS.

[52] Solomon IH, Chettimada S, Misra V, Lorenz DR, Gorelick RJ, Gelman BB (2020). White matter abnormalities linked to interferon, stress response, and energy metabolism gene expression changes in older HIV-positive patients on antiretroviral therapy. Mol Neurobiol.

[53] Fields JA, Spencer B, Swinton M, Qvale EM, Marquine MJ, Alexeeva A (2018). Alterations in brain TREM2 and Amyloid-beta levels are associated with neurocognitive impairment in HIV-infected persons on antiretroviral therapy. J Neurochem.

[54] Silva AC, Rodrigues BS, Micheletti AM, Tostes S, Meneses AC, Silva-Vergara ML (2012). Neuropathology of AIDS: an autopsy review of 284 cases from Brazil comparing the findings pre- and post-HAART (highly active antiretroviral therapy) and pre- and postmortem correlation. AIDS Res Treat.

[55] Sidova M, Tomankova S, Abaffy P, Kubista M, Sindelka R (2015). Effects of post-mortem and physical degradation on RNA integrity and quality. Biomol Detect Quantif.

[56] Koppelkamm A, Vennemann B, Lutz-Bonengel S, Fracasso T, Vennemann M (2011). RNA integrity in post-mortem samples: influencing parameters and implications on RT-qPCR assays. Int J Legal Med.

[57] Chaillon A, Gianella S, Dellicour S, Rawlings SA, Schlub TE, De Oliveira MF (2020). HIV persists throughout deep tissues with repopulation from multiple anatomical sources. J Clin Invest.

[58] Available from: http://lastgift.ucsd.edu/

[59] Vasquez JJ, Hunt PW (2019). Participating in human immunodeficiency virus cure research at the end of life. Clin Infect Dis.

[60] Perry KE, Taylor J, Patel H, Javadi SS, Mathur K, Kaytes A (2020). “[It] is now my responsibility to fulfill that wish:” clinical and rapid autopsy staff members’ experiences and perceptions of HIV reservoir research at the end of life. PLoS ONE.

[61] Maldarelli F (2020). The gift of a lifetime: analysis of HIV at autopsy. J Clin Invest.

[62] Rawlings SA, Chaillon A, Smith D, Gianella S (2021). Scale up rapid research autopsies for tissue immunology. Nature.

[63] Rawlings SA, Gianella S (2020). Tissue is the issue: how altruistic people with HIV are changing the HIV tissue reservoir landscape. Future Virol.

[64] Rawlings SA, Layman L, Smith D, Scott B, Ignacio C, Porrachia M (2020). Performing rapid autopsy for the interrogation of HIV reservoirs. AIDS.

[65] Ahrendsen JT, Filbin MG, Chi SN, Manley PE, Wright KD, Bandopadhayay P (2019). Increasing value of autopsies in patients with brain tumors in the molecular era. J Neurooncol.

[66] Alsop K, Thorne H, Sandhu S, Hamilton A, Mintoff C, Christie E, et al. A community-based model of rapid autopsy in end-stage cancer patients. Nat Biotechnol. 2016:1010–4. 10.1038/nbt.3674 27617737

[67] Bavi P, Siva M, Abi-Saab T, Chadwick D, Dhani N, Butany J (2019). Developing a pan-cancer research autopsy programme. J Clin Pathol.

[68] Hooper JE (2021). Rapid autopsy programs and research support: the pre- and post-COVID-19 Environments. AJSP Rev Rep.

[69] Morgello S, Gelman BB, Kozlowski PB, Vinters HV, Masliah E, Cornford M (2001). The National NeuroAIDS Tissue Consortium: a new paradigm in brain banking with an emphasis on infectious disease. Neuropathol Appl Neurobiol.

[70] Heithoff AJ, Totusek SA, Le D, Barwick L, Gensler G, Franklin DR, et al. The integrated National NeuroAIDS Tissue Consortium database: a rich platform for neuroHIV research. Database (Oxford). 2019;2019:bay134. 10.1093/database/bay134 PMC6323298 30624650

[71] Javadi SS, Mathur K, Concha-Garcia S, Patel H, Perry KE, Lo M (2021). Attitudes and perceptions of next-of-kin/loved ones toward end-of-life HIV cure-related research: a qualitative focus group study in Southern California. PLoS ONE.

[72] Zhang T, Gupta A, Frederick D, Layman L, Smith DM, Gianella S, et al. 3D visualization of immune cell populations in HIV-infected tissues via clearing, immunostaining, confocal, and light sheet fluorescence microscopy. J Vis Exp. 2021(171). 10.3791/62441 10.3791/62441 PMC10445482 34028448

[73] Tan Y-L, Yuan Y, Tian L (2020). Microglial regional heterogeneity and its role in the brain. Mol Psychiatry.

[74] Masuda T, Sankowski R, Staszewski O, Prinz M (2020). Microglia heterogeneity in the single-cell era. Cell Rep.

[75] De Scheerder MA, Vrancken B, Dellicour S, Schlub T, Lee E, Shao W (2019). HIV rebound is predominantly fueled by genetically identical viral expansions from diverse reservoirs. Cell Host Microbe.

[76] Sannier G, Dube M, Dufour C, Richard C, Brassard N, Delgado GG (2021). Combined single-cell transcriptional, translational, and genomic profiling reveals HIV-1 reservoir diversity. Cell Rep.

[77] Mangus LM, Dorsey JL, Laast VA, Hauer P, Queen SE, Adams RJ (2015). Neuroinflammation and virus replication in the spinal cord of simian immunodeficiency virus-infected macaques. J Neuropathol Exp Neurol.

[78] Graves MC, Fiala M, Dinglasan LA, Liu NQ, Sayre J, Chiappelli F (2004). Inflammation in amyotrophic lateral sclerosis spinal cord and brain is mediated by activated macrophages, mast cells and T cells. Amyotroph Lateral Scler Other Motor Neuron Disord.

[79] Schnell G, Joseph S, Spudich S, Price RW, Swanstrom R (2011). HIV-1 replication in the central nervous system occurs in two distinct cell types. PLoS Pathog.

[80] Gianella S, Kosakovsky Pond SL, Oliveira MF, Scheffler K, Strain MC, De la Torre A (2016). Compartmentalized HIV rebound in the central nervous system after interruption of antiretroviral therapy. Virus Evol.

[81] Ellis RJ, Gamst AC, Capparelli E, Spector SA, Hsia K, Wolfson T (2000). Cerebrospinal fluid HIV RNA originates from both local CNS and systemic sources. Neurology.

[82] Lustig G, Cele S, Karim F, Derache A, Ngoepe A, Khan K (2021). T cell derived HIV-1 is present in the CSF in the face of suppressive antiretroviral therapy. PLoS Pathog.

[83] Choi JY, Chaillon A, Oh JO, Ahn JY, Ann HW, Jung IY (2016). HIV migration between blood plasma and cellular subsets before and after HIV therapy. J Med Virol.

[84] Hendricks CM, Cordeiro T, Gomes AP, Stevenson M (2021). The interplay of HIV-1 and macrophages in viral persistence. Front Microbiol.

[85] Oliveira MF, Chaillon A, Nakazawa M, Vargas M, Letendre SL, Strain MC (2017). Early antiretroviral therapy is associated with lower HIV DNA molecular diversity and lower inflammation in cerebrospinal fluid but does not prevent the establishment of compartmentalized HIV DNA populations. PLoS Pathog.

[86] Sturdevant CB, Joseph SB, Schnell G, Price RW, Swanstrom R, Spudich S (2015). Compartmentalized replication of R5 T cell-tropic HIV-1 in the central nervous system early in the course of infection. PLoS Pathog.

[87] Nguyen LT, Schmidt HA, von Haeseler A, Minh BQ (2015). IQ-TREE: a fast and effective stochastic algorithm for estimating maximum-likelihood phylogenies. Mol Biol Evol.

[88] Giatsou E, Abdi B, Plu I, Desire N, Palich R, Calvez V (2020). Ultradeep sequencing reveals HIV-1 diversity and resistance compartmentalization during HIV-encephalopathy. AIDS.

[89] Srinivas N, Rosen EP, Gilliland WM, Kovarova M, Remling-Mulder L, De La Cruz G (2019). Antiretroviral concentrations and surrogate measures of efficacy in the brain tissue and CSF of preclinical species. Xenobiotica.

[90] Devanathan AS, Pirone JR, Akkina R, Remling-Mulder L, Luciw P, Adamson L, et al. Antiretroviral penetration across three preclinical animal models and humans in eight putative HIV viral reservoirs. Antimicrob Agents Chemother. 2019;64(1):e01639-19. 10.1128/AAC.01639-19 PMC7187610 31611355

[91] Joseph SB, Swanstrom R (2018). The evolution of HIV-1 entry phenotypes as a guide to changing target cells. J Leukoc Biol.

[92] Joseph SB, Arrildt KT, Sturdevant CB, Swanstrom R (2015). HIV-1 target cells in the CNS. J Neurovirol.

[93] Wang C, Schlub TE, Yu WH, Tan CS, Stefic K, Gianella S, et al. Landscape of HIV neutralization susceptibilities across tissue reservoirs. Clin Infect Dis. 2022;75(8):1342–50. 10.1093/cid/ciac164 PMC9555844 35234862

[94] Ratai EM, Bombardier JP, Joo CG, Annamalai L, Burdo TH, Campbell J (2010). Proton magnetic resonance spectroscopy reveals neuroprotection by oral minocycline in a nonhuman primate model of accelerated NeuroAIDS. PLoS ONE.

[95] Nakasujja N, Miyahara S, Evans S, Lee A, Musisi S, Katabira E (2013). Randomized trial of minocycline in the treatment of HIV-associated cognitive impairment. Neurology.

[96] Shiver JW, Fu TM, Chen L, Casimiro DR, Davies ME, Evans RK (2002). Replication-incompetent adenoviral vaccine vector elicits effective anti-immunodeficiency-virus immunity. Nature.

[97] Policicchio BB, Pandrea I, Apetrei C (2016). Animal models for HIV cure research. Front Immunol.

[98] Micci L, McGary CS, Paiardini M (2015). Animal models in HIV cure research. J Virus Erad.

[99] Fitzgerald DW, Janes H, Robertson M, Coombs R, Frank I, Gilbert P (2011). An Ad5-vectored HIV-1 vaccine elicits cell-mediated immunity but does not affect disease progression in HIV-1-infected male subjects: results from a randomized placebo-controlled trial (the Step study). J Infect Dis.

[100] Massanella M, Ignacio RAB, Lama JR, Pagliuzza A, Dasgupta S, Alfaro R (2021). Long-term effects of early antiretroviral initiation on HIV reservoir markers: a longitudinal analysis of the MERLIN clinical study. Lancet Microbe.

[101] Pinzone MR, VanBelzen DJ, Weissman S, Bertuccio MP, Cannon L, Venanzi-Rullo E (2019). Longitudinal HIV sequencing reveals reservoir expression leading to decay which is obscured by clonal expansion. Nat Commun.

[102] Bruner KM, Wang Z, Simonetti FR, Bender AM, Kwon KJ, Sengupta S, et al. A quantitative approach for measuring the reservoir of latent HIV-1 proviruses. Nature. 2019;566(7742):120–5. 10.1038/s41586-019-0898-8 PMC6447073 30700913

[103] Hiener B, Horsburgh BA, Eden JS, Barton K, Schlub TE, Lee E (2017). Identification of genetically intact HIV-1 proviruses in specific CD4(+) T cells from effectively treated participants. Cell Rep.

[104] Lu CL, Pai JA, Nogueira L, Mendoza P, Gruell H, Oliveira TY (2018). Relationship between intact HIV-1 proviruses in circulating CD4(+) T cells and rebound viruses emerging during treatment interruption. Proc Natl Acad Sci U S A.

[105] Dufour CRM, Pagliuzza A. Cattin A, Salinas T, Salahuddin S, Schinkel S, Costiniuk C, Jenabian M, Ancuta P, Routy J, Cohen E, Power C, Angel J, Chomont N. Expansion and extensive recirculation of HIV-infected cells in multiple organs. Conference on Retroviruses and Opportunistic Infections (CROI); Denver, CO: In Special Issue: Abstracts From the 2022 Conference on Retroviruses and Opportunistic Infections. Top Antiv Med. 2022;30(1s):137.

[106.•] Patro SC, Brandt LD, Bale MJ, Halvas EK, Joseph KW, Shao W (2019). Combined HIV-1 sequence and integration site analysis informs viral dynamics and allows reconstruction of replicating viral ancestors. Proc Natl Acad Sci U S A.

[107] Nguyen QD, Moodie EM, Forget MF, Desmarais P, Keezer MR, Wolfson C (2021). Health heterogeneity in older adults: exploration in the Canadian longitudinal study on aging. J Am Geriatr Soc.

[108] Moise N, Wood D, Cheung YKK, Duan N, Onge TS, Duer-Hefele J (2018). Patient preferences for personalized (N-of-1) trials: a conjoint analysis. J Clin Epidemiol.

[109] Kronish IM, Cheung YK, Shimbo D, Julian J, Gallagher B, Parsons F (2019). Increasing the precision of hypertension treatment through personalized trials: a pilot study. J Gen Intern Med.

[110] Iasonos A, O’Quigley J (2021). Randomised Phase 1 clinical trials in oncology. Br J Cancer.

[111] Cabarrou B, Sfumato P, Mourey L, Leconte E, Balardy L, Martinez A (2018). Addressing heterogeneity in the design of phase II clinical trials in geriatric oncology. Eur J Cancer.

[112] Hutter G, Nowak D, Mossner M, Ganepola S, Mussig A, Allers K (2009). Long-term control of HIV by CCR5 Delta32/Delta32 stem-cell transplantation. N Engl J Med.

[113] Gupta RK, Peppa D, Hill AL, Galvez C, Salgado M, Pace M (2020). Evidence for HIV-1 cure after CCR5Delta32/Delta32 allogeneic haemopoietic stem-cell transplantation 30 months post analytical treatment interruption: a case report. Lancet HIV.

[114] Simonetti FR, Sobolewski MD, Fyne E, Shao W, Spindler J, Hattori J (2016). Clonally expanded CD4+ T cells can produce infectious HIV-1 in vivo. Proc Natl Acad Sci U S A.

[115] Wagner TA, McLaughlin S, Garg K, Cheung CY, Larsen BB, Styrchak S (2014). HIV latency. Proliferation of cells with HIV integrated into cancer genes contributes to persistent infection. Science.

[116] Reid VL, McDonald R, Nwosu AC, Mason SR, Probert C, Ellershaw JE (2017). A systematically structured review of biomarkers of dying in cancer patients in the last months of life; an exploration of the biology of dying. PLoS ONE.

[117] Saul J, Hutchins E, Reiman R, Saul M, Ostrow LW, Harris BT (2020). Global alterations to the choroid plexus blood-CSF barrier in amyotrophic lateral sclerosis. Acta Neuropathol Commun.

[118] Kumar N, Singh A, Kulkarni RV (2015). Transcriptional bursting in gene expression: analytical results for general stochastic models. PLoS Comput Biol.

[119] Wang X, Park J, Susztak K, Zhang NR, Li M (2019). Bulk tissue cell type deconvolution with multi-subject single-cell expression reference. Nat Commun.

[120] Chen Z, Huang A, Sun J, Jiang T, Qin FX-F, Wu A (2017). Inference of immune cell composition on the expression profiles of mouse tissue. Sci Rep.

[121] Zoutman WH, Nell RJ, Versluis M, van Steenderen D, Lalai RN, Out-Luiting JJ (2017). Accurate quantification of T cells by measuring loss of germline T-cell receptor loci with generic single duplex droplet digital PCR assays. J Mol Diagn.

[122] Dube K, Gianella S, Concha-Garcia S, Little SJ, Kaytes A, Taylor J (2018). Ethical considerations for HIV cure-related research at the end of life. BMC Med Ethics.

[123] Kanazawa J, Gianella S, Concha-Garcia S, Taylor J, Kaytes A, Christensen C (2022). Ethical and practical considerations for HIV cure-related research at the end-of-life: a qualitative interview and focus group study in the United States. BMC Med Ethics.

[124] George LK. Research design in end-of-life research: state of science. Gerontologist. 2002;42 Spec No 3:86–98. 10.1093/geront/42.suppl_3.86 12415138

[125] Sandstrom TS, Burke Schinkel SC, Angel JB (2019). Medical assistance in death as a unique opportunity to advance human immunodeficiency virus cure research. Clin Infect Dis.

[126] Perry KE, Dube K, Concha-Garcia S, Patel H, Kaytes A, Taylor J (2020). “My death will not [be] in vain”: testimonials from last gift rapid research autopsy study participants living with HIV at the end of life. AIDS Res Hum Retroviruses.

[127] Dube K, Patel H, Concha-Garcia S, Perry KE, Mathur K, Javadi SS (2020). Perceptions of next-of-kin/loved ones about last gift rapid research autopsy study enrolling people with HIV/AIDS at the end of life: a qualitative interview study. AIDS Res Hum Retroviruses.

[128] Gianella S, Taylor J, Brown TR, Kaytes A, Achim CL, Moore DJ (2017). Can research at the end of life be a useful tool to advance HIV cure?. AIDS.

[129] Prakash K, Gianella S, Dube K, Taylor J, Lee G, Smith DM (2018). Willingness to participate in HIV research at the end of life (EOL). PLoS ONE.

[130] Karris MY, Dube K, Moore AA (2020). What lessons it might teach us? Community engagement in HIV research. Curr Opin HIV AIDS.

[131] Dube K, Perry KE, Mathur K, Lo M, Javadi SS, Patel H (2020). Altruism: scoping review of the literature and future directions for HIV cure-related research. J Virus Erad.

[132] Wilkins CH (2018). Effective engagement requires trust and being trustworthy. Med Care.

[133] Gianella S, Tsibris A, Barr L, Godfrey C (2016). Barriers to a cure for HIV in women. J Int AIDS Soc.

[134] Newton JD (2011). How does the general public view posthumous organ donation? A meta-synthesis of the qualitative literature. BMC Public Health.

